# Drug Delivery Vehicle—Teriflunomide Interactions Affect Treatment and Fluorine Magnetic Resonance in an Animal Model of Multiple Sclerosis

**DOI:** 10.3390/pharmaceutics18101225

**Published:** 2026-09-27

**Authors:** Xiang Hu, Nandita Saha, Yinhao Chen, Jason M. Millward, Andreas Oder, Keven Mallow, Stefanie Münchberg, Christian Prinz, Helmar Waiczies, Michael Rothe, Martin Neuenschwander, Marc Nazaré, Friedemann Paul, Thoralf Niendorf, Sonia Waiczies

**Affiliations:** 1 Berlin Ultrahigh Field Facility, Max-Delbrück-Center for Molecular Medicine in the Helmholtz Association (MDC), 13125 Berlin, Germany; xiang.hu@mdc-berlin.de (X.H.); nandita.saha@charite.de (N.S.); chen.yinhao@mdc-berlin.de (Y.C.); jason.millward@mdc-berlin.de (J.M.M.); stefanie.muenchberg@mdc-berlin.de (S.M.); thoralf.niendorf@mdc-berlin.de (T.N.); 2 Charité-Universitätsmedizin Berlin, 10117 Berlin, Germany; friedemann.paul@charite.de; 3 Experimental and Clinical Research Center (ECRC), A Joint Cooperation Between the Charité Medical Faculty and the Max-Delbrück Center for Molecular Medicine in the Helmholtz Association, 13125 Berlin, Germany; 4 Medicinal Chemistry, Leibniz-Forschungsinstitut für Molekulare Pharmakologie (FMP), 13125 Berlin, Germany; oder@fmp-berlin.de (A.O.); mallow@fmp-berlin.de (K.M.); neuenschwander@fmp-berlin.de (M.N.); nazare@fmp-berlin.de (M.N.); 5 SRH Fernhochschule—The Mobile University, 88499 Riedlingen, Germany; christian.prinz@mobile-university.de; 6 Tool Time GmbH, 10245 Berlin, Germany; helmar@waiczies.de; 7 Lipidomix GmbH, 13125 Berlin, Germany; michael.rothe@lipidomix.de

**Keywords:** teriflunomide, multiple sclerosis, experimental autoimmune encephalomyelitis, voluntary oral administration, drug-delivery vehicle, fluorine-19 magnetic resonance imaging, fluorine-19 magnetic resonance spectroscopy, tissue distribution, pharmacodynamics

## Abstract

**Background/Objectives**: Teriflunomide (TF) is a fluorinated oral therapy approved for relapsing-remitting multiple sclerosis that can be detected using fluorine-19 (^19^F) magnetic resonance (MR) methods. In preclinical studies, palatable drug delivery vehicles may facilitate voluntary oral administration and reduce procedure-related stress; however, they may also interact with the drug and alter its pharmacological and MR properties. We investigated whether sweetened milk (SM), compared with sucrose-containing carboxymethylcellulose (SCMC), affects the therapeutic efficacy, tissue distribution, and ^19^F MR signal of TF in experimental autoimmune encephalomyelitis (EAE), a mouse model of MS. **Methods**: Twenty-four female SJL/J mice received TF (30 mg/kg/day) in SM or SCMC, or the corresponding vehicle controls, by micropipette-guided drug administration (MDA) from day −2 to day 22. Clinical disease was monitored; hepatic TF was assessed ex vivo by ^19^F MR spectroscopy (MRS) and imaging (MRI) at 9.4 T; and brain TF was quantified by HPLC-MS. Formulation-dependent ^19^F MR spectral and MR relaxation properties were examined in vitro using phantoms containing TF in different vehicles, including DMSO, SM, SCMC, and serum. Intrinsic pharmacodynamic activity was evaluated by dihydroorotate dehydrogenase (DHODH) inhibition. **Results**: TF in SCMC reduced EAE severity during peak disease and delayed disease onset, whereas TF in SM produced no sustained therapeutic benefit. Hepatic ^19^F MR signals were approximately twofold higher with SCMC than with SM, and brain TF concentrations were 4.8-fold higher. Phantom studies revealed formulation-dependent changes in chemical shift, spectral linewidth, and the MRI relaxation time T_2_, including a 65-fold shortening in CMC + SM relative to CMC alone. SM did not reduce the intrinsic DHODH inhibitory potency of TF, indicating that the diminished in vivo efficacy was not attributable to impaired pharmacodynamic activity. **Conclusions**: As an oral delivery vehicle, sweetened milk altered TF therapeutic efficacy, as well as ^19^F MR signal and tissue distribution in the EAE model. Palatable vehicles used for voluntary oral treatment should therefore be carefully selected and validated, as vehicle–drug interactions may alter tissue exposure, confound ^19^F MR detection and, more importantly, compromise therapeutic efficacy.

## 1. Introduction

Multiple sclerosis (MS) is a chronic inflammatory disease of the central nervous system (CNS) that is the leading cause of neurological disability in young adults [1,2]. Experimental autoimmune encephalomyelitis (EAE) is the most widely used animal model of MS. The model is induced by immunization with myelin-derived self-antigens, triggering a T-cell-mediated autoimmune response against the CNS [3].

Teriflunomide (TF) is an approved oral drug for relapsing–remitting (RR) MS. It blocks the mitochondrial enzyme dihydroorotate dehydrogenase (DHODH), thereby limiting de novo pyrimidine synthesis required for the proliferation of activated T cells [4,5,6]. Its therapeutic efficacy was first investigated in EAE, including proteolipid protein (PLP)-induced EAE in SJL/J mice [7,8,9,10]. The SJL/J model is widely used to study immunomodulatory therapies for RRMS because PLP-induced EAE in this strain follows a relapsing-remitting disease course resembling the clinical pattern of RRMS [11]. It is therefore particularly appropriate for evaluating treatments such as TF that are indicated for relapsing forms of MS.

TF contains a trifluoromethyl (CF_3_) group, and we previously demonstrated that its levels can be measured by fluorine-19 (^19^F) magnetic resonance (MR): we detected TF in EAE mouse brains using ^19^F MR spectroscopy (MRS) [12]. The property of fluorinated drugs to be detected by ^19^F MR methods enables the pharmacological effects of drug treatments to be investigated alongside their MR-based reporting characteristics [12,13]. Unlike conventional proton (^1^H) MR, ^19^F MR benefits from the absence of endogenous fluorine in biological tissues, enabling direct detection of fluorinated compounds [14,15]. Accordingly, ^19^F MR methods offer a non-invasive means of assessing the biodistribution of fluorinated drugs. While ^19^F MRS enables the detection and quantification of fluorinated drugs such as TF [12], ^19^F MR imaging (MRI) provides spatially resolved information on the tissue distribution [13].

Studying the biodistribution of orally administered drugs may also help identify interactions with dietary components such as milk that might reduce gastrointestinal absorption, thereby limiting systemic and tissue exposure [16]. Gastrointestinal absorption is a central determinant for oral drug delivery, a most commonly used administration route [17]. It is governed by anatomical, physiological, and formulation-related factors that collectively determine oral bioavailability [18,19]. Interaction between orally administered drugs and dietary components may also be relevant in animal models. Oral administration is widely used and preferred to other routes (intravenous, intraperitoneal, subcutaneous) because it is non-invasive and closely resembles the clinical route of treatment [20]. The interaction concern becomes particularly important when oral gavage with a comparatively inert suspending vehicle, such as carboxymethylcellulose (CMC), is replaced by voluntary administration in a palatable food-based vehicle such as sweetened milk [21], which may compromise drug bioavailability.

Micropipette-guided drug administration (MDA) for voluntary intake is becoming a popular alternative drug administration method, as animals learn to drink from the pipette tip [21,22]. Voluntary oral administration represents a refinement in accordance with the 3Rs—Replacement, Reduction, and Refinement [23]—by reducing the repeated restraint, handling, and procedure-related stress associated with oral gavage [21,22,24,25]. This is particularly relevant in EAE, where stress may alter immune responses and disease severity [26].

However, the vehicle used to promote voluntary intake may itself interact with the drug and alter its absorption, bioavailability, and therapeutic efficacy. Sweetened milk (SM) is widely used for MDA because of its high palatability [21,22], but it is a compositionally complex vehicle containing casein micelles that could associate with hydrophobic compounds and modify their physicochemical behavior [27]. For example, nanoencapsulation of vitamin D_3_ in casein micelles improved its stability while maintaining its bioavailability [28,29]. This demonstrates that milk proteins can influence the stability and delivery of associated compounds. Such interactions are compound-dependent and may therefore either enhance or impair drug availability.

The molecular environment strongly influences the ^19^F MR properties of fluorinated drugs, as demonstrated for TF [12], and its fluorinated derivatives di-CF_3_-TF and SF_5_-TF [30], as well as the MS drug siponimod [13]. In serum, protein association markedly shortens the MR relaxation times T_2_ and T_2_*, resulting in rapid signal decay and spectral line broadening [12,13,30,31]. Similar effects have been observed in liver tissue for siponimod, highlighting the need to consider tissue-dependent relaxation when interpreting ^19^F MR measurements of drug distribution [13]. Both TF and siponimod are more than 99% bound to serum proteins [32], which likely contributes to the pronounced shortening of their transverse relaxation times in biological matrices.

These observations prompted us to investigate whether the protein-containing sweetened-milk vehicle used in MDA could likewise influence the pharmacological availability and ^19^F MR properties of teriflunomide (TF). In the present study, we compared sweetened milk (SM) with a protein-free sucrose–carboxymethylcellulose (SCMC) vehicle for voluntary micropipette-guided drug administration (MDA) of TF in SJL/J EAE mice to determine whether vehicle composition influences therapeutic efficacy, tissue distribution, and ^19^F MR detectability. CMC is a protein-free suspending agent that is widely used as an oral gavage vehicle in EAE treatment studies [9,12,33,34]. Because CMC alone was insufficiently palatable for voluntary oral consumption in the MDA paradigm, it was supplemented with sucrose to produce SCMC as a protein-free alternative to SM. We employed a multimodal approach combining clinical assessment, ex vivo ^19^F MRS/MRI at 9.4 T, HPLC-MS quantification, in vitro MR relaxometry, and DHODH inhibition assays to assess vehicle-dependent effects on therapeutic efficacy and tissue distribution of TF, as well as its ^19^F MR properties.

## 2. Materials and Methods

### 2.1. In Vivo Experiments

#### 2.1.1. Ethics Statement

All procedures were approved by the Animal Welfare Department of the State Office for Health and Social Affairs Berlin (LAGeSo; License G0123/21) and conducted in accordance with European Directive 86/609/EEC.

#### 2.1.2. Preparation of Drug Delivery Vehicles for In Vivo Application

TF was synthesized at the Berlin Leibniz-Forschungsinstitut für Molekulare Pharmakologie (FMP) [25]. SM was prepared by diluting condensed milk 1:4 (Brand: Nestlé Milchmädchen, Vevey, Switzerland) with deionized water to a standard working concentration containing 1.9% milk protein, 11% sucrose, and 2.75% lactose. For the TF_SM_ group, TF was added to SM and homogenized. SCMC was composed of carboxymethylcellulose (0.5% *w*/*v*), Tween-80 (0.1% *v*/*v*), and sucrose (10% *w*/*v*) dissolved in deionized water. For the TF_SCMC_ group, TF was suspended in the SCMC and vortexed. All formulations were stored at 4 °C and vortexed before oral administration.

#### 2.1.3. Drug Treatment and Experimental Autoimmune Encephalomyelitis

Twenty-four female SJL/J mice (8–10 weeks old; Janvier Labs, Le Genest-Saint-Isle, France) were housed three per cage under specific pathogen-free, standardized conditions (including enrichment with nesting material/shelters), a 12-h light/dark cycle, and food/water available ad libitum. Following a two-week quarantine and acclimatization, all mice underwent 14 days of habituation to micropipette-guided drug administration (MDA) using their respective drug-free vehicles SM or SCMC, to minimize administration-related stress and ensure reliable consumption of the complete dose during the subsequent treatment experiments (Figure 1A).

Mice were randomly assigned to four groups (n = 6 per group) upon arrival in their holding rooms: two untreated vehicle groups receiving either SM (Ø_SM_) or SCMC (Ø_SCMC_), and two TF-treated groups receiving TF in SM (TF_SM_) or TF in SCMC (TF_SCMC_). An a priori sample-size calculation was performed based on effect sizes observed in previous preclinical EAE studies of teriflunomide [7,8,9,10,12].

TF treatment was administered once daily at 8:30 a.m. by voluntary MDA (Figure 1A,B). TF-treated mice received body weight-adjusted volumes of TF_SM_ or TF_SCMC_ to deliver a daily dose of 30 mg/kg, whereas vehicle-control mice received a fixed volume of 50 µL of the corresponding vehicle. During each dosing session, every mouse was dosed individually and continuously observed until the complete administered volume had been consumed before being returned to the cage. Treatment began two days before EAE induction (day −2) and continued through day 22 (Figure 1B).

EAE was induced on day 0 by subcutaneous immunization with 250 µg PLP_139–151_ peptide (Pepceuticals, Leicester, UK) emulsified in 100 µL Complete Freund’s Adjuvant (BD Difco, Franklin Lakes, NJ, USA) supplemented with 800 µg *Mycobacterium tuberculosis* H37RA (List Biological Laboratories, Campbell, CA, USA) [12]. Each mouse additionally received 220 ng pertussis toxin (Hooke Laboratories, Lawrence, MA, USA) by intraperitoneal injection on days 0 and 2 after immunization [12]. Body weight and neurological impairment were assessed up to three times daily. A standardized EAE scoring system was used: 0.5 = impaired righting reflex or partial tail paresis, 1.0 = complete tail paralysis, 1.5 = additional hindlimb paresis, 2.0 = additional hindlimb paralysis, and 2.5 = additional severe motor deficits, including involvement of a forelimb. The total daily EAE score was calculated by summing the scores assigned to the individual neurological deficits. The primary outcome measure was EAE clinical disease severity, assessed by the daily EAE score. Animals reaching the predefined humane endpoints, based on neurological severity and the extent and duration of body-weight loss, were withdrawn from the experiment in accordance with animal-welfare requirements.

### 2.2. MR Hardware

All ex vivo and in vitro MR measurements were performed on a 9.4 T pre-clinical MR scanner (Bruker Biospin, Ettlingen, Germany). Ex vivo MR imaging and spectroscopy of EAE tissue were performed using a cryogenically cooled 2-channel transceiver ^19^F quadrature RF surface probe (ID = 20 mm, Cryoprobe, Figure 1E, Bruker, Fällanden, Switzerland) [35] and a ^1^H transmit/receive RF coil (ID = 72 mm, Bruker Biospin, Ettlingen, Germany). In vitro phantom MR relaxometry measurements were acquired with an in-house-built dual-tuned ^19^F/^1^H mouse-head volume RF coil (ID = 16 mm, Figure 4B) [36]. All scans were conducted at room temperature, and the acquisition environment was kept constant within each measurement.

### 2.3. Ex Vivo Experiments with EAE Tissue

#### 2.3.1. Sample Collection

On day 22, mice were anesthetized via intraperitoneal injection of ketamine (500 mg/kg) and xylazine (10 mg/kg). The depth of anesthesia was confirmed by the absence of the toe-pinch nociceptive withdrawal reflex. Without recovery from anesthesia, the animals were humanely euthanized by transcardial perfusion with 20 mL of ice-cold phosphate-buffered saline. Following perfusion, the liver and brain were immediately collected (Figure 1C). Liver samples were immersion-fixed in 4% paraformaldehyde for 48 h at 4 °C for ex vivo ^19^F MR measurements. Brain tissues were snap-frozen on dry ice and stored at −20 °C until quantitative analysis of TF by HPLC-MS.

#### 2.3.2. High-Performance Liquid Chromatography Mass Spectrometry (HPLC-MS)

Ex vivo brain tissues were homogenized in ice-cold buffer. Proteins were precipitated by adding cold acetonitrile, followed by centrifugation (e.g., 15,000× *g* for 10 min at 4 °C) to recover the clarified supernatants. Following filtration, samples were injected into an Agilent 1290 UHPLC system (Agilent Technologies, Santa Clara, CA, USA) equipped with a Kinetex C18 column (Phenomenex, Torrance, CA, USA) (Figure 1D). Chromatographic separation was achieved using a water/acetonitrile gradient supplemented with ammonium acetate. Detection was performed on an Agilent 6470 triple-quadrupole mass spectrometer operating in negative electrospray ionization (ESI-) mode. Multiple Reaction Monitoring (MRM) was used to monitor the characteristic TF transitions at *m*/*z* 269.2 → 160.0 and 269.2 → 82.0. TF quantification was conducted by Lipidomix GmbH (Berlin, Germany) using the same validated analytical procedure as previously described [12].

#### 2.3.3. MR Measurements of Ex Vivo Tissue

MR measurements of ex vivo liver tissues were performed using the ^19^F Cryoprobe and ^1^H volume RF coil (see above, Figure 1E). Tissue samples were embedded in 2% agarose within 5 mL tubes and positioned against the inner wall of the tube facing the surface of the ^19^F Cryoprobe, thereby minimizing the distance between the tissue and the coil. The agarose maintained this orientation and prevented the tissue from shifting away from the coil during the MR measurements.

Anatomical ^1^H images of the ex vivo liver samples were first acquired using a Rapid Acquisition with Relaxation Enhancement (RARE) MR technique: echo time (TE) = 49.2 ms, repetition time (TR) = 2616.6 ms, number of averages (Avg) = 2, receiver bandwidth (BW_receiver_) = 75 kHz, acquisition time (TA) = 10 min, echo spacing = 8.2 ms, rare factor = 8, slices = 24, slice thickness = 1 mm, field of view (FoV) = 24 × 24 mm^2^, image matrix size = 192 × 192. The ^1^H images were used to verify the position of the liver sample within the FoV. TF signal intensity was then assessed by ^19^F MR spectroscopy (MRS). ^19^F spectra were acquired for all ex vivo liver samples using the ^19^F Cryoprobe and a block pulse for excitation: TR = 400 ms, Avg = 9000, TA = 1 h, acquisition points = 1024, acquisition bandwidth = 200 kHz, flip angle (FA) = 48°. Subsequently, three-dimensional (3D) ^19^F MR images of the ex vivo liver were acquired using Ultrashort Echo-Time (UTE): TE = 0.142 ms, TR = 10 ms, Avg = 820, TA = 4 h, FA = 14°, image matrix = 24 × 24 × 24, FoV = 64 × 64 × 64 mm^3^, Gaussian excitation pulse, BW_receiver_ = 200 kHz, 2-fold radial undersampling. Two-dimensional sections reconstructed from the ^19^F UTE datasets were overlaid on the corresponding anatomical ^1^H images [13,30].

### 2.4. In Vitro MR Relaxometry Experiments

#### 2.4.1. Phantom Preparation

A series of in vitro phantoms was prepared in 2.5 mL syringes (Figure 4A) to evaluate the impact of drug delivery vehicles on the ^19^F MR properties of TF. The tested vehicles included dimethyl sulfoxide (DMSO), DMSO + SM (sweetened milk), DMSO + sucrose, CMC, CMC + SM, CMC + sucrose, and human serum. Each syringe phantom contained TF at a concentration of 2.7 mg/mL that was thoroughly vortexed before MR measurements.

#### 2.4.2. MR Measurements of Phantoms 

MR relaxometry measurements with the in vitro phantoms were performed using the in-house-built dual-tuned ^19^F/^1^H mouse-head volume RF coil (see above, Figure 4B). ^19^F MRS was used to determine the chemical shift and longitudinal relaxation time (T_1_) of TF. Measurements were performed using a single-pulse free-induction decay (FID) sequence using 23 TRs, ranging from 100 to 10,000 ms. TRs were increased in 100 ms increments from 100 to 1500 ms, followed by TRs of 2000, 2500, 3000, 4000, 5000, 6000, 8000, and 10,000 ms. The representative ^19^F spectrum of TF was acquired at TR = 1000 ms. A Carr–Purcell–Meiboom–Gill (CPMG) pulse sequence with 25 echo times (TEs) was used to measure the transverse relaxation time (T_2_). For DMSO-based phantoms, the echo spacing (ES) was 40 ms, and the TR was 5000 ms. For CMC-based phantoms, the ES was 10.6 ms and the TR was 5000 ms. For the serum phantom, the ES was 2.8 ms and the TR was 2000 ms. The number of averages was kept constant at 50 for all phantom measurements [12,30].

### 2.5. MR Data Analysis

Processing of all MRS and MRI data was performed in MATLAB 2021b (The MathWorks, Natick, MA, USA), using a previously described processing pipeline [13].

#### 2.5.1. Processing of ^19^F MRS Data and Spectral Quantification

All chemical shifts were referenced to trichlorofluoromethane (CFCl_3_). The FIDs from the ex vivo MRS measurements were processed by removing the digital filter delay and zero-filling before Fourier transformation. Spectra acquired with the unloaded ^19^F Cryoprobe were used to characterize the coil-derived ^19^F artifact signal. The artifact contribution was estimated by fitting complex Voigt functions and subsequently subtracted from the spectra [13,37]. Complex Voigt peaks were then fitted separately to each receive channel for phase correction, after which signals from the individual channels were averaged to obtain the combined spectrum. A Voigt peak was fitted to the resulting spectrum to quantify the TF signal intensity and extract the peak area, center frequency, and full width at half maximum (FWHM). The combined spectrum was calculated as the mean of the spectra acquired from the two receive channels. Noise was determined from a background region of the combined spectrum and estimated as the mean of the standard deviations of the noise from the two receive channels. The peak signal-to-noise ratio (pSNR) was then calculated as the ratio of the maximum real spectral intensity of the combined spectrum to the estimated background noise [13]. Identical processing steps were applied to all ex vivo samples to ensure comparability.

#### 2.5.2. Processing of ^19^F UTE-3D and ^1^H RARE MR Images

Raw ^19^F UTE k-space data acquired using a radial trajectory, together with the corresponding trajectory information, were extracted from Bruker data files and reconstructed [38]. Radial spokes were averaged across repetitions and reconstructed using a non-uniform fast Fourier transform [39]. Images from the individual receiver channels were subsequently combined using the sum-of-squares method, followed by background noise-based thresholding to suppress the false-positive fluorine signal [13]. The reconstructed ^19^F volumes, with a matrix of 24 × 24 × 24, were upsampled to match the 192 × 192 × 192 matrix of the corresponding ^1^H anatomical images [40]. ^19^F signals exceeding the noise threshold were then displayed as colour-coded masks overlaid on the grayscale ^1^H liver anatomy images.

#### 2.5.3. Processing of ^19^F MR Relaxometry Data

T_1_ and T_2_ relaxation times were estimated by fitting the ^19^F signal intensity data obtained from ^19^F MRS using MATLAB’s non-linear least-squares optimization function lsqcurvefit. T_1_ relaxation times were determined by fitting signal intensities acquired at different repetition times (TRs) using the mono-exponential longitudinal relaxation equation, $S(TR)\;\propto \;1\;-\;exp\left(-\frac{TR}{T1}\right)$ [41]. T_2_ relaxation times were determined by fitting signal intensities acquired at different echo times (TEs) using the mono-exponential transverse relaxation equation, $S(TE)\;\propto \;exp\left(-\frac{TE}{T2}\right)$ [41]. The accuracy of the fit for both T_1_ and T_2_ relaxation times was evaluated using the coefficient of determination (R^2^), calculated as $R^{2}\;=\;1\;-\;\left(\frac{{SS}_{res}}{{SS}_{tot}}\right)$, where SS_res_ is the residual sum of squares, and SS_tot_ is the total sum of squares [42].

### 2.6. DHODH Enzyme Inhibition Assay

The inhibitory activity of TF in different formulation conditions was assessed using a colorimetric DHODH assay, as previously described [12,30]. TF stock solutions were prepared in DMSO, SM, DMSO + SM, serum, CMC, and SCMC and diluted to comparable working concentrations. DHODH activity was quantified using a 2,6-dichlorophenolindophenol (DCIP)-based readout at 620 nm in 384-well plates containing recombinant human DHODH and the required assay-buffer components. Absorbance was recorded over 60 min at 28 °C. For IC_50_ determination, serial TF dilutions were prepared by acoustic dispensing for non-CMC formulations and by conventional pipetting for CMC-containing formulations. Reaction rates were normalized to matched vehicle controls. Dose–response curves were fitted by nonlinear regression using the four-parameter logistic model LL.4 of R package drc. The response differences were calculated from the differences in response between the highest and the lowest doses, determined from the fitted curve. IC_50_ values were derived from the inflection point of the fitted dose–response curve. The Hill coefficient describes the steepness of the fitted dose–response curve around the IC_50_ concentration and was also determined using the R package drc.

### 2.7. Statistical Analysis

All statistical analyses were performed using R (version 4.3.1). As EAE clinical scores are discrete, ordinal measurements, non-parametric analysis was used. EAE clinical scores were compared among the four groups using the Kruskal–Wallis test for each day, followed by Dunn’s post hoc test with Bonferroni correction for multiple comparisons. Correction for multiple comparisons across days was done with the Benjamini–Hochberg false discovery method. Time to disease onset was analyzed using the log-rank test. Given the small group sizes, the underlying distributions could not be reliably characterized by formal normality tests. Two-group comparisons of ex vivo liver ^19^F MRS peak SNR and HPLC-derived ex vivo brain TF concentrations were analyzed using the non-parametric Mann–Whitney U test. Statistical significance was defined as *p* < 0.05.

## 3. Results

### 3.1. Role of Sweetened Milk on TF Treatment in EAE

Vehicle-treated SJL/J mice receiving either SM (Ø_SM_) or SCMC (Ø_SCMC_) developed a typical EAE disease course. Clinical signs appeared at approximately days 9–10 post-immunization, and mean clinical scores peaked between days 12 and 13 (Figure 2A and Figure S1). There were no significant differences between the vehicle groups (Ø_SM_ and Ø_SCMC_) on any day (Figure 2B), and both reached a cumulative disease incidence of 100% (Figure 2C), indicating that neither vehicle influenced EAE induction.

**Figure 2:**
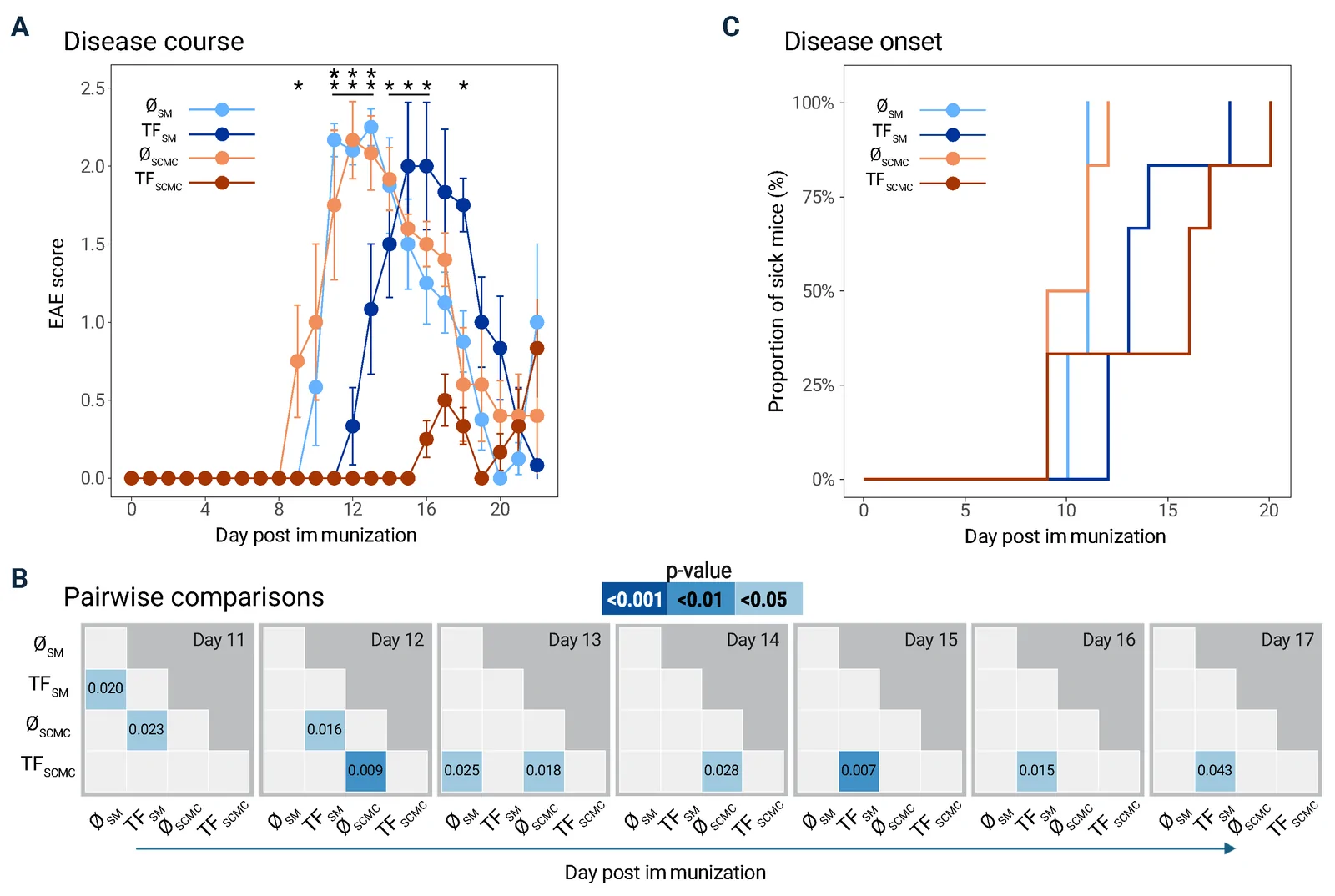
Impact of oral delivery vehicles on the therapeutic efficacy of teriflunomide (TF) in EAE. (**A**) Clinical EAE scores over time in SJL/J mice receiving TF in sweetened milk (TF_SM_) or sucrose-containing CMC (TF_SCMC_), together with the corresponding vehicle-only control groups (Ø_SM_ and Ø_SCMC_; n = 6 per group). Data are presented as mean ± standard error of the mean (SEM). Asterisks indicate time points at which significant differences among the four groups were detected by the Kruskal–Wallis test (nominal *p* < 0.05). (**B**) Pairwise comparisons of clinical EAE scores among the four groups were analyzed for each time point. Displayed in the heatmaps are statistically significant comparisons observed from Day 11 to Day 17, with the corresponding adjusted *p*-values from the Dunn’s multiple-comparisons test with Bonferroni correction; *p* < 0.05). (**C**) Cumulative proportion of mice developing clinical EAE, defined as an EAE score ≥ 0.5, over time in the four experimental groups. * *p* < 0.05; ** *p* < 0.01; *** *p* < 0.001.

Consistent with the previously reported therapeutic efficacy of TF in EAE, including in the SJL/J model [8,12], we observed that TF administered in SCMC (TF_SCMC_) attenuated disease severity (Figure 2A). Clinical scores remained low during the expected peak phase and were significantly lower than those of the vehicle-treated groups, particularly between days 11 and 17 post-immunization (Figure 2A: asterisks refer to the nominal *p*-values from the Kruskal–Wallis test; Figure 2B shows post-hoc pairwise comparisons). After correction for multiple comparisons across days, group differences remained significant for days 11–13. Time-to-onset analysis showed a significant overall difference in disease onset among the four groups (log-rank, *p* = 0.0048, Figure 2C).

In contrast, TF administered in SM (TF_SM_) did not produce a pronounced reduction in disease severity, despite a slight delay in disease onset (Figure 2C). Once clinical signs appeared, scores increased to levels comparable to those observed in the vehicle-treated groups (Figure 2A), indicating that TF administered in sweetened milk (TF_SM_) did not attenuate subsequent disease severity.

### 3.2. Effect of Sweetened Milk on TF Distribution in Liver and Brain

To determine whether the formulation vehicle influenced TF tissue distribution, we combined ex vivo ^19^F MRI/MRS of the liver with HPLC-MS quantification of TF in brain tissue (Figure 3). In our previous study, we found that ex vivo TF concentrations in the brain were below the detection limit of ^19^F MR [12]. Therefore, cerebral TF levels were assessed exclusively by HPLC-MS, whereas the higher TF accumulation in the liver enabled compound-specific detection by ^19^F MRS and spatial localization by ^19^F MRI. Together, these complementary methods provided convergent evidence of formulation-dependent differences in TF tissue distribution.

**Figure 3:**
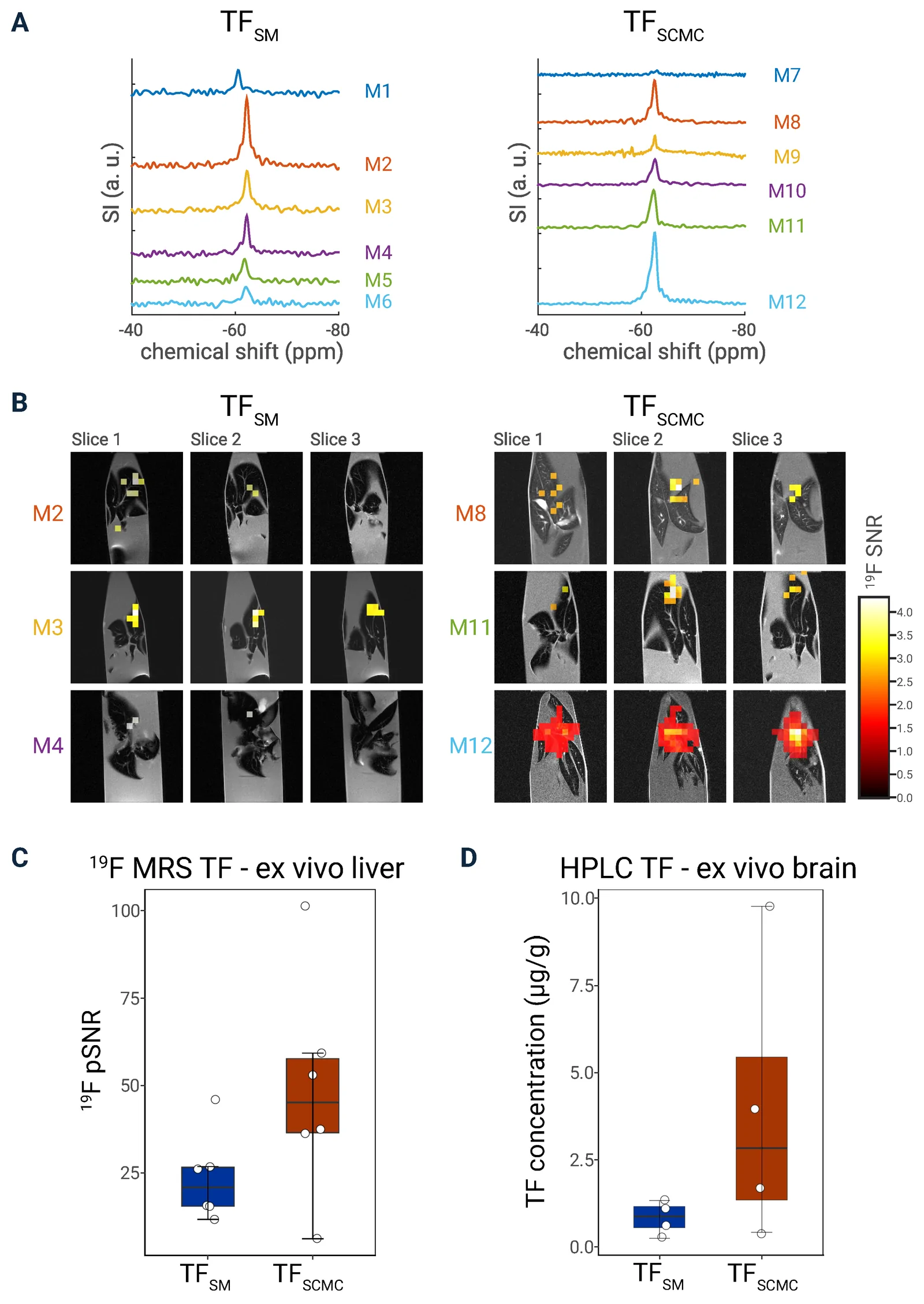
^19^F MRS/MRI and HPLC-MS of teriflunomide (TF) in ex vivo tissues from mice treated with TF_SM_ and TF_SCMC_. (**A**) Individual ^19^F MR spectra of ex vivo liver samples from TF_SM_ (**left**)- and TF_SCMC_ (**right**)-treated mice. Each spectrum represents one animal. The TF-associated ^19^F signals are displayed using a common signal-intensity (S.I.) scale across all animals in both treatment groups, with spectra vertically offset for clarity. (**B**) Ex vivo UTE-3D ^19^F MRI overlaid on ex vivo ^1^H liver images from the three mice with the highest ^19^F MR peak SNR in each treatment group, as determined from panel A: M2, M3, and M4 in the TF_SM_ group (**left**) and M8, M11, and M12 in the TF_SCMC_ group (**right**). (**C**) Quantification of TF ^19^F MRS peak SNR in ex vivo liver samples (n = 6 per group). (**D**) TF concentrations measured by HPLC-MS in ex vivo brain samples (n = 4 per group). Data are presented as individual values with box-and-whisker plots indicating the median and interquartile range.

Consistent with the expected resonance of TF, ex vivo ^19^F MRS detected a single resonance at approximately −61 ppm in all liver samples from both TF-treated groups (n = 6 per group, TF_SM_ animal IDs: M1–M6 and TF_SCMC_ animal IDs: M7–M12, Figure 3A). However, the signal intensity differed between the two formulations. The TF_SM_ group showed a lower peak SNR than the TF_SCMC_ group (Figure 3A,C).

The spatially resolved ex vivo ^19^F MRI findings were consistent with the ^19^F MRS results. The individual ^19^F MR spectra were plotted using the same signal-intensity scale across all animals in each group (Figure 3A), enabling direct comparison of the ^19^F MR signal amplitudes. Based on the ^19^F peak SNR, the three mice with the highest signals in each treatment group were selected for ex vivo ^19^F MRI: M2, M3, and M4 from the TF_SM_ group and M8, M11, and M12 from the TF_SCMC_ group (Figure 3B). This selection was made solely for visualization of the spatial ^19^F signal distribution and was not used for quantitative group comparison. Livers selected from the TF_SM_-treated group displayed weaker and less extensive ^19^F signals than the selected TF_SCMC_ livers, which generally showed stronger and more widely distributed ^19^F signals (Figure 3B). Quantitative comparison of the hepatic ^19^F signal was based on all six animals per group. The TF_SM_ group showed a lower peak SNR than the TF_SCMC_ group (23.52 ± 5.15 versus 48.84 ± 12.90, respectively; n = 6 per group; Figure 3C).

A similar formulation-dependent difference was observed in the brain. HPLC-MS analysis revealed an approximately 4.8-fold lower TF concentration in the TF_SM_ group than the TF_SCMC_ group (0.83 ± 0.25 versus 3.95 ± 1.90; n = 4 per group; Figure 3D). These results corroborate the liver MR findings, showing that SM delivery leads to reduced TF levels in both the liver and the brain.

### 3.3. Influence of Sweetened Milk on ^19^F MR Properties of TF

To determine whether the formulation environment influenced the ^19^F MR characteristics of TF, we measured its chemical shift in ppm, spectral linewidth, and longitudinal (T_1_) and transverse (T_2_) relaxation times in phantoms containing TF in different drug-delivery vehicles (Figure 4A) using the in-house-built ^19^F/^1^H volume RF coil (Figure 4B).

All samples exhibited a single detectable ^19^F resonance. However, the chemical shift and linewidth of the TF resonance, as well as T_2_ relaxation properties, depended on the vehicle (Figure 4C, Table 1).

The ^19^F resonance was centered at approximately −58 ppm in the DMSO-based formulations, including DMSO alone, DMSO with SM, and DMSO with sucrose. In contrast, the resonance was shifted to approximately −61 ppm in all CMC-based formulations and in serum (Figure 4C, Table 1). This approximately 3-ppm difference indicates that TF experienced distinct local physicochemical environments in the DMSO- and CMC-based formulations.

The full width at half maximum (FWHM) values also varied among the formulations (Figure 4C, Table 1). Within the DMSO-based group, the linewidths were relatively similar, ranging from 96 Hz in DMSO with sucrose to 160 Hz in DMSO with SM, compared with 128 Hz in DMSO alone. TF in CMC and SCMC exhibited the narrowest resonances, with FWHM values of 79 and 74 Hz, respectively. In contrast, the addition of SM to CMC broadened the ^19^F peak to 374 Hz, representing an approximately 4.7-fold increase compared with CMC alone. Thus, SM had only a moderate effect on linewidth in the DMSO formulation but caused pronounced line broadening when combined with CMC. TF in serum also displayed a broad resonance, with a FWHM of 313 Hz as previously reported [12].

**Figure 4:**
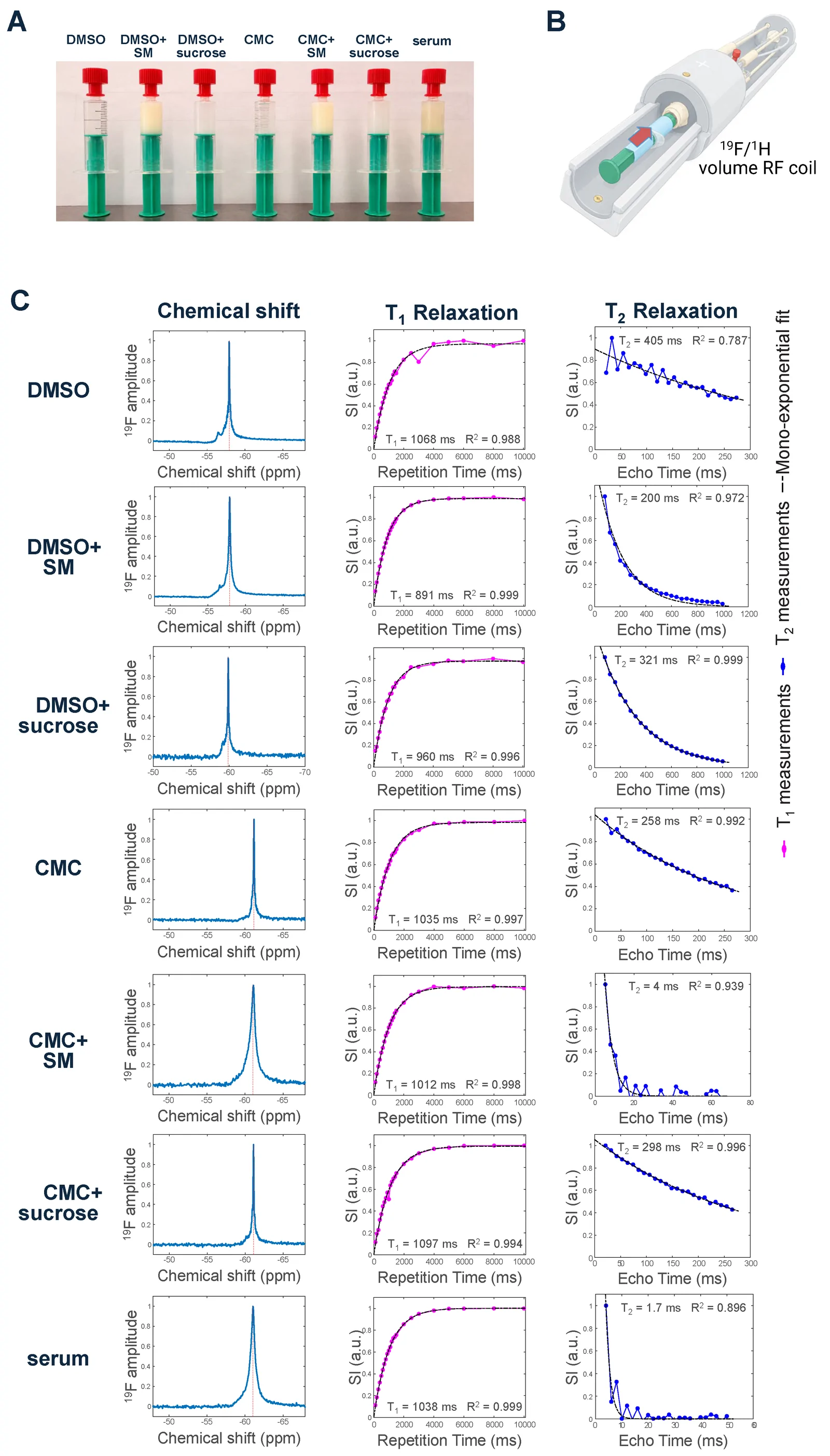
In vitro phantom preparation, measurement setup, and ^19^F MR characterization of teriflunomide (TF) under different formulation conditions. (**A**) Photograph of TF phantoms prepared in dimethyl sulfoxide (DMSO), DMSO + sweetened milk (SM), DMSO + sucrose, carboxymethylcellulose (CMC), CMC + SM, CMC + sucrose, and serum. (**B**) Schematic representation of the phantom measurement setup using a dual-tuned ^19^F/^1^H volume RF coil. The illustration was generated from an original photograph of the setup using AI-assisted image transformation. (**C**) Representative ^19^F spectra (TR = 1000 ms), T_1_ relaxation curves, and T_2_ relaxation curves of TF at 2.7 mg/mL and room temperature in each formulation. ^19^F MR spectra were acquired with a repetition time of 1000 ms. T_1_ and T_2_ values were determined by mono-exponential fitting of signal intensity as a function of repetition time and echo time, respectively. Symbols indicate measured data, and solid lines represent the corresponding fits.

**Table 1:** ^19^F MR spectral and relaxation parameters of TF in different conditions.

MR Property	DMSO	DMSO+ SM	DMSO+ Sucrose	CMC	CMC+ SM	SCMC	Serum
Chemical shift (ppm) *	−58	−58	−58	−61	−61	−61	−61
FWHM (Hz) ^#^	128	160	96	79	374	74	313
T_1_ relaxation (ms)	1068	891	960	1035	1012	1097	1038
T_2_ relaxation (ms)	405	200	321	258	4	298	1.7

* Chemical shift values estimated based on visual peak assignment (±0.1 ppm; no spectral fitting applied). ^#^ FWHM values were obtained from Lorentzian fits.

T_1_ relaxation times of TF showed comparatively modest variation across formulations (Figure 4C, Table 1), ranging from 891 ms in DMSO + SM to 1097 ms in SCMC. Values in DMSO, CMC, CMC + SM, SCMC and serum were similar, at 1068, 1035, 1012, 1097, and 1038 ms, respectively.

By contrast, the T_2_ relaxation time of TF varied markedly across formulations (Figure 4C, Table 1). TF in DMSO alone exhibited a T_2_ of 405 ms. The addition of sucrose (DMSO + sucrose) moderately reduced T_2_ to 321 ms, and the addition of SM (DMSO + SM) shortened it to 200 ms. Among the CMC-based formulations, T_2_ remained in the hundreds of milliseconds for CMC alone and SCMC, at 258 and 298 ms, respectively. However, combining CMC with SM caused a pronounced reduction in T_2_ to 4 ms, approaching the very short T_2_ of 1.7 ms observed in serum, as previously reported [12].

### 3.4. Sweetened Milk Does Not Impair DHODH Inhibition by TF

Because TF administered in sweetened milk showed reduced therapeutic efficacy in EAE compared with TF administered in SCMC (Figure 2), we investigated whether SM directly impaired the pharmacological activity of TF. The inhibitory activity of TF against recombinant dihydroorotate dehydrogenase (DHODH), its primary molecular target, was therefore assessed under different vehicle conditions (Figure 5; Table 2).

TF produced well-defined concentration–response curves in DMSO, DMSO supplemented with SM, and SM alone. The corresponding IC_50_ values were comparable and remained within the sub-micromolar range at 0.62, 0.38, and 0.51 µM, respectively. The response differences were 92%, 117%, and 117%, with Hill coefficients of 1.47, 1.04, and 1.26, respectively. Thus, neither the addition of SM to DMSO nor the use of SM alone reduced the potency or maximal inhibitory response of TF. TF in serum also retained submicromolar inhibitory activity, with an IC_50_ of 0.22 µM, a response difference of 121%, and a Hill coefficient of 0.73.

In contrast, the CMC-containing conditions produced atypical concentration–response profiles. In CMC alone, the curve could not be fitted reliably. Therefore, no valid IC_50_ value lying within the range of measured doses or response difference or Hill coefficient could be determined. In SCMC, curve fitting yielded an IC_50_ of 0.03 µM, which was below the range of measured doses. The response difference was reduced to 54.1%, and the Hill coefficient was only 0.33, indicating a shallow and incomplete concentration–response relationship. The fitted IC_50_ under this condition should therefore be interpreted cautiously and cannot be considered a robust estimate of TF potency.

## 4. Discussion

This study demonstrates that the composition of the oral drug-delivery vehicle is an important determinant of the therapeutic efficacy, tissue distribution, and ^19^F MR detection of teriflunomide (TF) in the SJL/J EAE model. To our knowledge, it is the first to integrate therapeutic monitoring in EAE, tissue drug quantification with ex vivo ^19^F MRI/MRS and HPLC-MS, in vitro MR drug relaxometry, and target-enzyme inhibition. This approach allowed us to distinguish among the effects of drug delivery vehicles on pharmacological activity and ^19^F MR drug properties.

Previous EAE studies of MS therapeutics, including teriflunomide and siponimod, have used CMC as the sole oral delivery vehicle [9,12,33,34]. However, voluntary administration by MDA requires a palatable vehicle such as sweetened milk, introducing a compositionally distinct matrix that may interact with the drug. Such vehicles are frequently regarded as pharmacologically inert. Our findings demonstrate that vehicle selection can albeit influence therapeutic outcomes and should therefore be considered an integral component of preclinical study design. Consistent with the previously reported therapeutic efficacy of TF in EAE, including in SJL/J mice [7,8,9,10], we observed delayed disease onset and reduced clinical scores in mice treated with TF administered in SCMC (TF_SCMC_). Differences in EAE scores were most pronounced on days 11–13, which survived correction for multiple comparisons across time; differences at other time-points were more modest. In comparison, vehicle-treated SJL/J mice receiving SCMC (Ø_SCMC_) developed the expected EAE disease course. In contrast, TF administered in sweetened milk (TF_SM_) produced only a transient delay in the appearance of clinical signs and did not result in a sustained reduction in disease severity compared to vehicle-treated mice receiving SM (Ø_SM_). These findings indicate that the efficacy of orally administered TF is dependent on the vehicle in which it is delivered, specifically SM versus SCMC in this study.

The difference in pharmacological efficacy was accompanied by differences in tissue TF levels. Ex vivo ^19^F MRS showed an approximately twofold higher TF peak SNR in the liver of mice treated with TF in SCMC, than those treated in sweetened milk. In addition, spatially resolved ^19^F MRI demonstrated stronger and more extensive hepatic signals following TF administration in SCMC (TF_SCMC_) compared to SM (TF_SM_). HPLC-MS provided independent evidence of a difference in tissue distribution, with an approximately 4.8-fold higher mean TF concentration in the brains of mice receiving TF_SCMC_ than in those receiving TF_SM_. The concordant direction of the liver ^19^F MRI/MRS and brain HPLC-MS findings supports a biologically relevant formulation-dependent difference in TF exposure. Dietary-based administration vehicles cannot necessarily be assumed to be inert. When certain drugs such as temozolomide, montelukast, and mesalazine are mixed with dietary vehicles, including milk, the altered dose delivery and chemical stability could result in clinically relevant underexposure [43,44]. These studies illustrate how apparently convenient dietary-based vehicles may reduce the amount of active compound ultimately available for absorption. The present findings extend this general principle to TF by showing that administration in SM was associated with both reduced tissue exposure and reduced therapeutic efficacy. More broadly, food may also influence the physicochemical characteristics of the drugs, including rate of dissolution, solubility, and permeability; consequently, it will affect their oral bioavailability and therapeutic efficacy [45]. Food–drug interactions are well established for certain orally administered therapeutics; for example, the calcium content of milk can substantially reduce the gastrointestinal absorption of tetracyclines by forming poorly absorbable chelates [46]. Although administering TF in a small volume of SM to mice is not equivalent to a standardized fed-state study, these mechanisms provide a plausible framework for understanding how SM could have affected TF dissolution, gastrointestinal release, absorption, or first-pass disposition. The present experiments were not designed to identify the specific process responsible, and dedicated pharmacokinetic and gastrointestinal studies will therefore be required. Our findings apply specifically to the diluted sweetened condensed-milk formulation tested here; whole milk or lactose-free milk may differ in composition and could consequently affect TF differently.

In addition to influencing tissue exposure, the formulation altered the ^19^F MR spectral and relaxation properties of TF. The approximately 3 ppm chemical shift difference between the DMSO-based and CMC-based formulations is consistent with previous observations for TF [12]. This likely reflects their different physicochemical environments, which may affect the electronic shielding of the CF_3_ group. Variations in solvation and non-covalent interactions, including hydrogen bonding, can alter the electronic environment of fluorinated groups and thereby affect their ^19^F chemical shifts [47]. Beyond these chemical-shift differences, formulation-dependent effects were most pronounced for T_2_ relaxation time and spectral linewidth, whereas T_1_ varied by less than approximately 1.2-fold across formulations. In DMSO, sweetened milk shortened T_2_ by >2-fold and broadened the linewidth by c. 1.3-fold. By contrast, adding sucrose to DMSO shortened T_2_ by only c. 1.2-fold and reduced the linewidth by c. 25%. The effect of milk was stronger in CMC, where it shortened T_2_ by approximately 65-fold and broadened the linewidth nearly 5-fold. In contrast, TF formulated in sucrose-containing CMC retained long T_2_ values and narrow linewidths comparable to CMC alone. This suggests that the observed transverse relaxation changes were attributable to milk components. The shortened T_2_ and spectral broadening observed in serum (150-fold shortening) is consistent with previous reports describing transverse relaxation shortening associated with drug-serum protein binding [12,13,30,31]. The combination of T_2_ shortening and spectral broadening observed in the SM-containing and serum samples is indicative of reduced molecular mobility and/or interactions between TF and components of the surrounding physicochemical environment. Such pronounced T_2_ shortening does not necessarily imply a paramagnetic or superparamagnetic relaxation mechanism. Changes in molecular mobility and exchange between different physicochemical environments can enhance transverse dephasing of the ^19^F signal, thereby shortening T_2_ and contributing to spectral broadening. Potential mechanisms include transient binding to proteins or lipids, aggregation, and the presence of heterogeneous physicochemical microenvironments [48]. The present MR measurements cannot distinguish among these mechanisms. While high-resolution microscopy could help identify aggregates where their presence is uncertain, it would not determine the contribution of aggregation to the observed relaxation changes. In the present study, TF was not completely solubilized in the SM- and CMC-containing formulations and particulate/aggregated material was expected. Importantly, pronounced transverse relaxation shortening was also observed for TF in serum and, as previously demonstrated for siponimod, persists in ex vivo tissue [13]. In the present study, however, the ex vivo TF signal was insufficient for reliable tissue relaxation measurements comparable to those previously performed for siponimod [13]. The multiple mechanisms that could alter drug relaxation were not directly examined, and the phantom findings should therefore not be interpreted as proof of a specific interaction with milk proteins. Nevertheless, they demonstrate that the same TF concentration can exhibit markedly different ^19^F MR properties depending on the vehicle.

When interpreting the ex vivo MR findings, weaker hepatic ^19^F signals observed after TF_SM_ administration cannot be attributed exclusively to a lower tissue TF concentration. ^19^F MR signal intensity also depends on MR properties and molecular mobility, apart from pharmacology and tissue distribution. The presence of SM-related constituents could reduce TF detectability through T_2_ shortening, independent of concentration. Therefore, the lower TF signal may reflect a combination of reduced hepatic exposure and less favorable ^19^F MR properties. However, the independently quantified brain HPLC-MS results support an actual reduction in tissue exposure following SM administration.

To determine whether the formulation-dependent differences in therapeutic efficacy were associated with changes in the intrinsic pharmacodynamic activity of TF, its inhibitory effect on recombinant DHODH was assessed under the different formulation conditions. Comparable sub-micromolar IC_50_ values were obtained in DMSO, SM, and DMSO supplemented with SM, indicating that sweetened milk did not impair TF-mediated inhibition of DHODH. The CMC-containing conditions yielded atypical concentration–response curves with restricted response ranges and unreliable curve fitting. The atypical profiles likely reflect matrix-related interference with the in vitro assay. These potentially involving factors such as viscosity, incomplete mixing, altered compound accessibility, or interference with the assay readout. Because these possibilities were not examined experimentally, the data do not permit attribution to a specific mechanism and should not be interpreted as evidence that CMC increases or decreases the intrinsic potency of TF. What these findings could demonstrate is that SM does not directly impair TF-mediated inhibition of DHODH. The reduced therapeutic efficacy of TF administered in SM is therefore unlikely to result from a loss of intrinsic target-inhibitory activity and is more consistent with formulation-dependent differences in drug exposure or bioavailability.

An important strength of this study is the integration of biological, analytical, and MR imaging and spectroscopy readouts to assess the impact of drug delivery vehicles on the efficacy and spatial distribution of therapies. EAE phenotyping established the formulation-dependent therapeutic response, while HPLC-MS provided direct quantification of TF in brain tissue. Ex vivo ^19^F MRI/MRS assessed hepatic detection and spatial distribution, phantom measurements characterized vehicle-dependent relaxation behavior, and the DHODH assay evaluated intrinsic target inhibition. This multimodal approach allowed us to distinguish among three related but non-equivalent parameters: pharmacological activity, tissue exposure, and MR detectability. Although this study was performed in female SJL/J mice with EAE, vehicle-dependent effects on drug exposure may also occur with other orally administered small molecules. However, the magnitude of such effects is likely to depend on the compound, formulation, species, and disease context and should not be directly extrapolated to humans.

This study also has some limitations. First, the relatively small group sizes limited statistical power, although the effects were observed using two different methods in two different organs. Second, tissue measurements were obtained at a single terminal time point, and plasma pharmacokinetic parameters, including C_max_ and AUC, were not measured. Therefore, the present data do not describe the temporal pharmacokinetic profile of TF or allow a complete assessment of formulation-dependent differences in systemic bioavailability. Additionally, HPLC-MS measurements were unavailable for all tissues, limiting complete validation of the MR findings. Third, phantom systems cannot fully reproduce the biochemical complexity of gastrointestinal contents or biological tissues. Finally, TF was not detectable by ^19^F MR in perfused brain tissue despite measurable concentrations by HPLC-MS. This finding highlights the limited sensitivity of current ^19^F MR methods at low tissue drug concentrations and illustrates a broader challenge for theranostic studies. Pharmacologically relevant drug exposure may occur below the threshold required for direct MR detection, complicating quantitative comparisons between drug imaging and pharmacological measurements. Previous studies have shown that the sensitivity and detection limits of ^19^F MRI and MRS depend not only on fluorine concentration but also on compound-specific relaxation properties, spectral linewidth, hardware performance, and acquisition parameters [49,50,51,52,53,54]. Studies of fluorinated MS therapeutics have further demonstrated that the biological environment can markedly alter ^19^F MR detectability [12,13].

Future studies should determine whether similar vehicle-dependent effects occur with other directly detectable fluorinated small-molecule drugs and should systematically investigate the relationships between formulation composition, tissue drug concentration, relaxation properties, and ^19^F MR detectability. Quantitative chemical analysis in matched tissues would help distinguish true differences in drug exposure from differences in MR visibility. Tissue-specific MR relaxation measurements could additionally support relaxation-dependent correction of the ^19^F MR signal, although for TF the ex vivo signal was insufficient for reliable T_2_/T_2_* determination. Such correction would therefore require improved ^19^F sensitivity or higher tissue ^19^F signal. These considerations emphasize that ^19^F MR signal intensity cannot be interpreted solely as a measure of tissue drug concentration, particularly when the biological environment substantially alters drug relaxation properties.

## 5. Conclusions

This study identifies that the oral delivery vehicle is an important determinant of therapeutic efficacy, tissue distribution, and ^19^F MR detection for TF. Compared with SCMC, administration in SM was associated with reduced therapeutic efficacy in SJL/J EAE, lower TF concentration in brain tissue, and weaker hepatic ^19^F MR signals. Because TF retained its DHODH-inhibitory activity in the presence of SM, the reduced therapeutic response is more likely related to vehicle-dependent effects on drug delivery and bioavailability than to loss of intrinsic pharmacological activity. Collectively, these findings establish drug delivery vehicle selection as an integral component of preclinical research on fluorinated drugs. More broadly, the multimodal framework presented here provides a systematic approach for evaluating formulation-dependent effects on therapeutic efficacy, biodistribution, and ^19^F MR detectability.

## Figures and Tables

**Figure 1:**
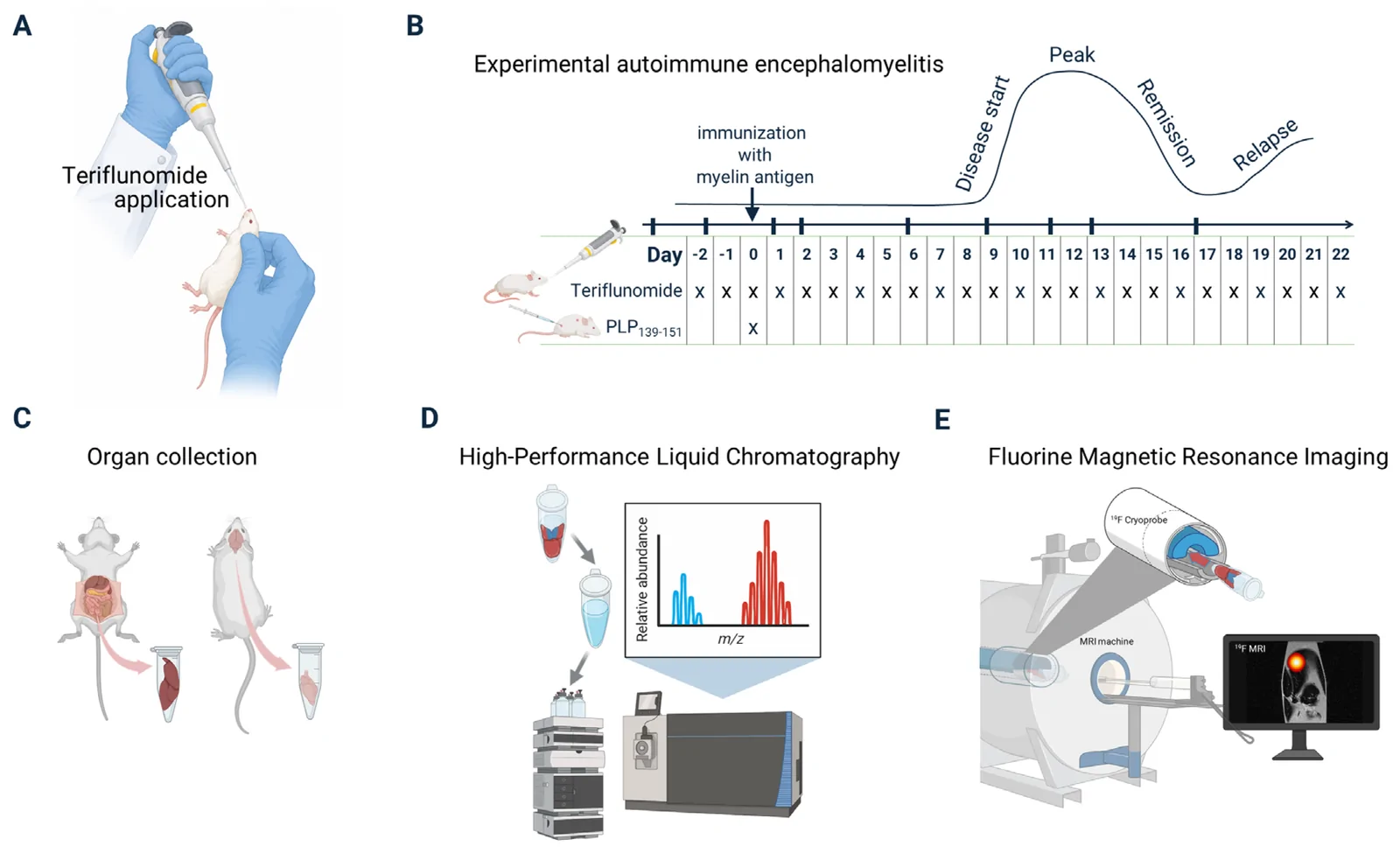
Experimental workflow and analytical methods. (**A**) Voluntary oral administration of teriflunomide (TF) was performed using micropipette-guided drug administration (MDA) without oral gavage. The illustration was generated from an original photograph using AI-assisted image transformation. (**B**) Experimental autoimmune encephalomyelitis (EAE) was induced in SJL/J mice by immunization with PLP_139–151_ peptide on Day 0. Daily TF treatment began two days before immunization and continued through Day 22 post-immunization. Clinical disease progression was monitored throughout. (**C**) Organs (liver and brain) were collected at the experimental endpoint for ex vivo analyses. (**D**) Schematic overview of the high-performance liquid chromatography–mass spectrometry (HPLC-MS) workflow used for TF quantification. (**E**) Schematic illustration of the ex vivo ^19^F magnetic resonance imaging (MRI) setup. Tissue samples were positioned under the ^19^F cryoprobe for MR acquisition. All panels were created in BioRender, with panel A additionally based on an AI-assisted transformation of an original photograph.

**Figure 5:**
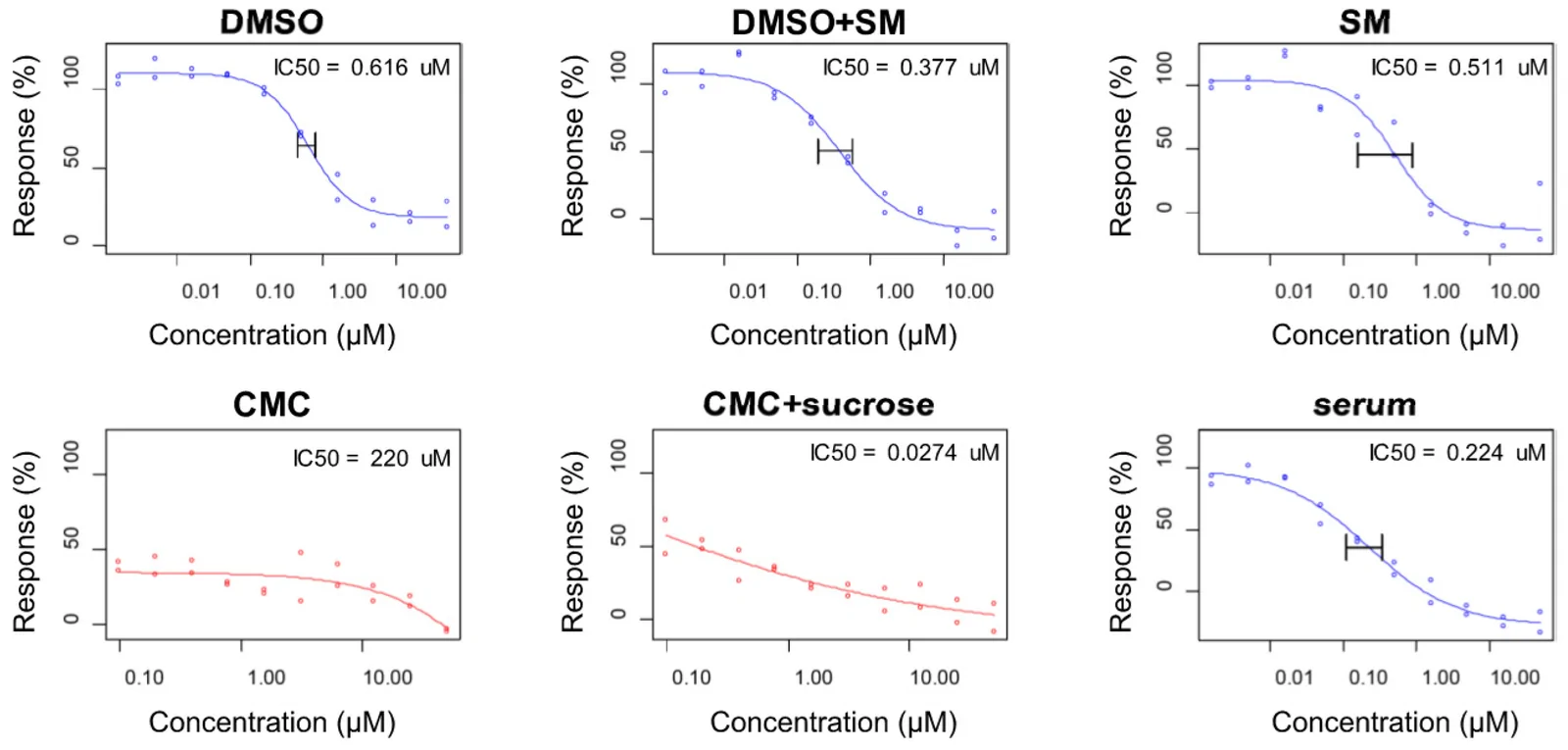
In vitro DHODH inhibitory potency of teriflunomide (TF) under different formulation conditions. Dose–response curves of TF measured in DMSO, DMSO + SM, SM, CMC, SCMC, and serum. The response (in %, from 0% indicating no enzyme activity to 100% indicating full enzyme activity) is plotted against the concentration of TF in µM. The assay was performed in technical duplicate in one biological replicate (n = 1). The horizontal black bar indicates the 95% confidence interval of the IC_50_ value. The calculated IC_50_ for each formulation condition are shown above the corresponding curve.

**Table 2:** In vitro DHODH inhibitory potency of TF under different conditions.

Condition	IC_50_ (µM)	95% Confidence Intervals	Response Difference (%)	Hill Coefficient
DMSO	0.62	0.45–0.78	92	1.47
DMSO + SM	0.38	0.19–0.56	117	1.04
SM	0.51	0.16–0.87	117	1.26
CMC	N.A.*	-	-	-
SCMC	0.03	−0.22–0.28	54.1	0.33
serum	0.22	0.11–0.34	121	0.73

* IC_50_ could not be reliably determined because the dose–response curve failed to produce a valid fit.

## Data Availability

The data that support the findings of this study are available from S.W. and X.H. upon reasonable request.

## Supplementary Materials

The following supporting information can be downloaded at: https://www.mdpi.com/article/10.3390/pharmaceutics18101225/s1, Figure S1: Individual animal-level EAE clinical scores over the experimental course. Longitudinal EAE clinical scores are shown for each individual animal in the four experimental groups.

## Abbreviations

The following abbreviations are used in this manuscript:

^19^F	fluorine-19
ANOVA	Analysis of Variance
AUC	area under the curve
CF_3_	trifluoromethyl
C_max_	maximum concentration
CMC	carboxymethylcellulose
CNS	central nervous system
CPMG	Carr-Purcell-Meiboom-Gill
DHODH	dihydroorotate dehydrogenase
DMSO	dimethyl sulfoxide
ES	echo spacing
EAE	Experimental Autoimmune Encephalomyelitis
FID	free-induction decay
FWHM	full width at half maximum
HPLC-MS	High-Performance Liquid Chromatography Mass Spectrometry
IC_50_	half-maximal inhibitory concentration
MDA	micropipette-guided drug administration
MR	magnetic resonance
MRI	MR imaging
MRS	MR spectroscopy
MS	multiple sclerosis
Ø_SCMC_	vehicle EAE group receiving SCMC
Ø_SM_	vehicle group receiving SM
RARE	Rapid Acquisition with Relaxation Enhancement
SCMC	sucrose–carboxymethylcellulose
SM	sweetened milk
T_1_	longitudinal relaxation time
T_2_	transverse relaxation time
TA	acquisition time
TE	echo time
TF	teriflunomide
TF_SCMC_	treatment with TF added to CMC + sucrose
TF_SM_	treatment with TF added to SM
TR	repetition time
UTE	Ultrashort Echo-Time
