# Supplementary material for: Drug Delivery Vehicle—Teriflunomide Interactions Affect Treatment and Fluorine Magnetic Resonance in an Animal Model of Multiple Sclerosis
Source: Pharmaceutics. 2026 Sep 27;18(10):1225. doi: 10.3390/pharmaceutics18101225 (PMC13708660; doi:10.3390/pharmaceutics18101225)
Supplement: Supplementary file 1 [file pharmaceutics-18-01225-s001.zip › pharmaceutics-4527344-supplementary.pdf]

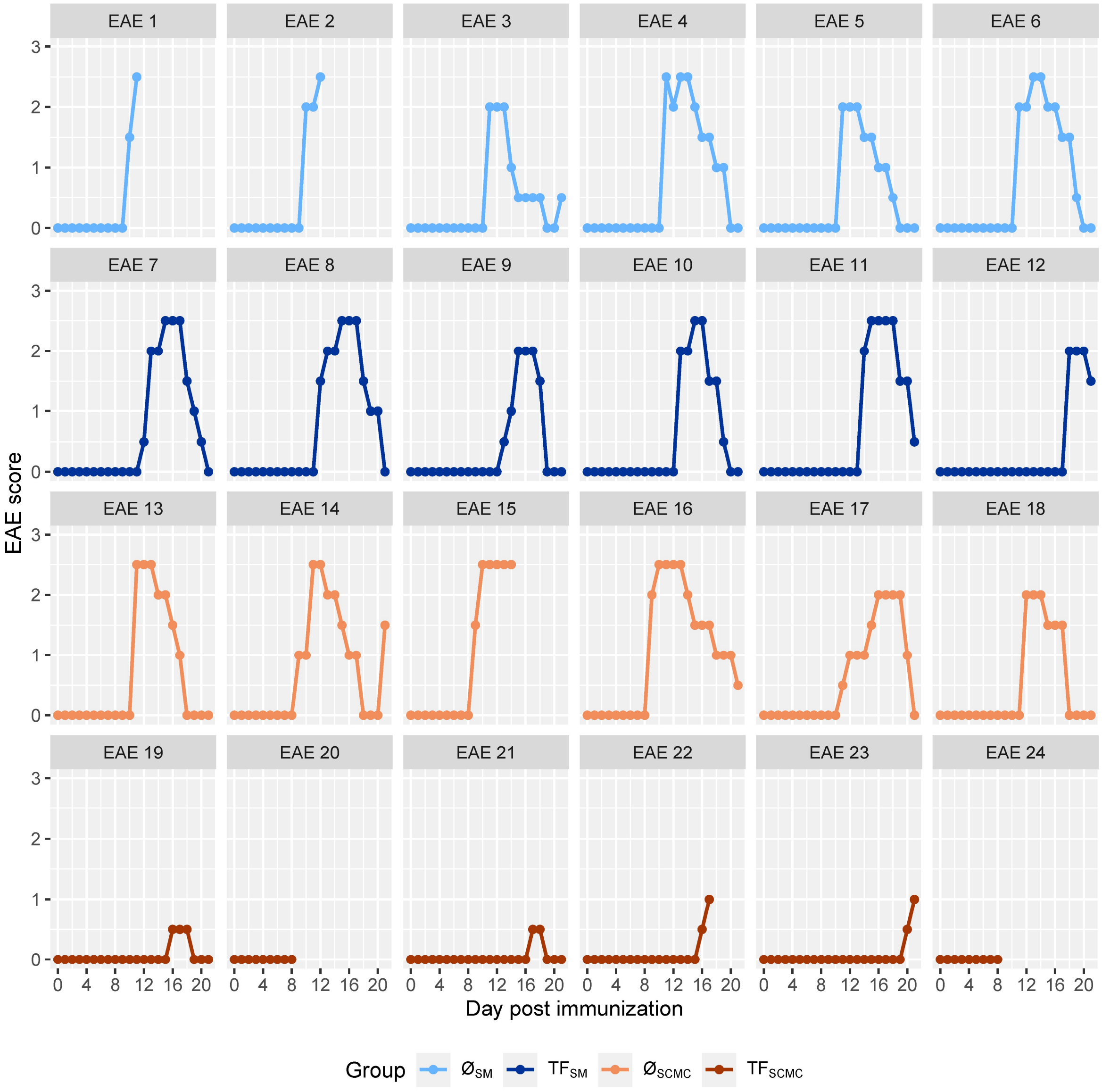

Figure S1. Individual animal-level EAE clinical scores over the experimental course. Longitudinal EAE clinical scores are shown for each individual animal in the four experimental groups.
